# Numerical simulation study of gas-solid two-phase flow characteristics in borehole sampling of coal cuttings

**DOI:** 10.1371/journal.pone.0337343

**Published:** 2025-12-04

**Authors:** Sheng Xue, Lei Zhang, Xiaoliang Zheng, Chuanyin Gao, Shilong Liu

**Affiliations:** 1 State Key Laboratory of Safe Mining of Deep Coal and Environment Protection, Anhui University of Science and Technology, Huainan, Anhui, China; 2 Joint National-Local Engineering Research Center for Safety and Precise Coal Mining, Anhui University of Science and Technology, Huainan, Anhui, China; 3 School of Safety Science and Engineering, Anhui University of Science and Technology, Huainan, Anhui, China; 4 School of Public Safety and Emergency Management, Anhui University of Science and Technology, Hefei, Anhui, China; Maria Curie-Sklodowska University: Uniwersytet Marii Curie-Sklodowskiej, POLAND

## Abstract

During the process of coal sampling through drilling and determining the gas content, the movement characteristics of coal particles within the borehole will affect the exposure time of the collected coal samples. Therefore, studying the movement characteristics of coal particles within the borehole is of great significance. This study employs CFD-DEM to simulate the transport dynamics of Rosin-Rammler distributed coal cuttings during pneumatic sampling, quantifying the effects of drill rod rotation (0–200 rpm) and particle size. Key findings reveal: (1) Axial migration velocity increases significantly with rotational speed, with 0.5–1 mm fine particles accelerating by 39% to 3.9 m s^-1^ at 100 rpm compared to static condition, though an optimal threshold exists at 150 rpm; (2) Rod rotation induces vortical flow fields, where bit geometry impedes coarse particles (>1 mm) in blade zones, while 0.5–1 mm particles migrate at velocities higher than 2–3 mm fractions under different rotational speeds; (3) Mass flow rate at the orifice doubles with speed (reaching 0.06 kg s^-1^ at 200 rpm), while static condition reduces efficiency by 50%. The observed significant velocity differentiation depending on particle size implies that using narrowly graded samples (e.g., obtained with adjacent sieve sizes such as 3–4 mm) could potentially improve the accuracy of lost gas content estimation by minimizing variations in particle transport history.

## Introduction

Coal and gas outburst incidents inflict substantial damage in Chinese underground mining operations [1–3]. Research confirms coal seam gas is a critical factor in coal and gas outbursts [4,5]. Because indirect gas content measurement methods are time-consuming, the direct method prevails in Chinese practice [6,7]. This direct method involves: (1) collecting borehole-orifice coal samples, (2) sealing samples in a coal container, and (3) quantifying gas content based on container desorption kinetics [8,9]. The direct method categorizes gas content into four components: lost gas (unmeasurable desorption before container sealing), desorbable gas, residual gas, and non-desorbable gas. Lost gas quantification relies on extrapolated desorption profiles [6]. Measurement inaccuracies primarily originate from lost gas estimation errors [5,6].

**Fig 1:**
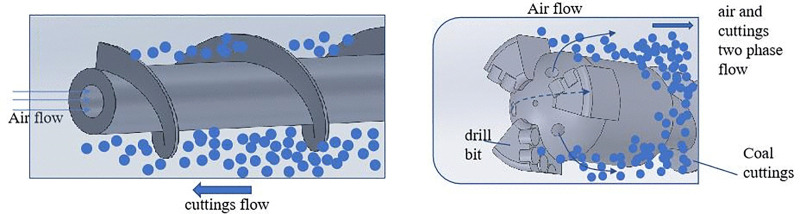
Characteristics of drill rod and drill cuttings transport in the borehole during sampling.

Coal cuttings collection at the borehole orifice for gas content measurement has a mixed particle size [10,11]. Borehole transit durations exhibit particle size dependency, inducing differential atmospheric exposure time in recovered samples. These temporal disparities constitute one of the error sources in lost gas quantification [12]. Compressed air and rod rotation drive cuttings migration within the borehole, with Fig 1 schematizing the rod-cuttings motion framework. Characterization of borehole cuttings transport dynamics remains essential for ensuring coal seam gas measurement accuracy.

Previous investigations of borehole coal cuttings transport primarily addressed particle size distributions and monodisperse particle behavior. Kang Jianning’s sampling experiments demonstrated extended transport durations for 10 mm cuttings versus 3 mm fractions under identical pneumatic pressure [13]. Zhang et al. analyzed Rosin-Rammler-distributed drill cuttings in tunnel environments, identifying gravitational settling of coarse fractions near the orifice while fine cuttings exhibited extended aerodynamic transport [14,15]. Conversely, experimental characterization of in-borehole migration dynamics remains limited owing to observational constraints. Advancements in computational modelling methodologies have enabled significant advances in pneumatic conveying simulations [16–20].

In pneumatic conveying simulations, flow regimes were classified as dilute-phase or dense-phase based on solid-phase volume fraction [16,21]. Dilute-phase analysis focuses on continuous phase effects on discrete particles, whereas dense-phase modelling requires bidirectional phase interaction. The CFD-DEM (Computational Fluid Dynamics-Discrete Element Method) methodology effectively characterises dense-phase particulate transport, including particle kinematics and inter-particle collisions [18,22].

The CFD-DEM methodology has been extensively applied across multiple domains, including agricultural engineering [23–27], chemical processing [19,28–31], pneumatic conveying [32,33], pharmaceutical manufacturing [34,35], and geohazard mitigation [36,37]. In drilling operations, this technique has enabled a comprehensive investigation of borehole coal cuttings transport—a characteristic dense-phase flow regime—yielding significant research outcomes [36,38]. For instance, Zhang et al. employed CFD-DEM analysis to examine drill rod rotation effects during vacuum-assisted sampling, revealing three key findings: rotational kinematics accelerate particulate transport velocities within flow conduits; an optimal rotational threshold exists; and operational gas-solid ratios consistently underperform theoretical values [39].

Although drilling cuttings transport dynamics in boreholes have been investigated, the impact of particle size distribution on sampling integrity during pneumatic drilling remains inadequately characterized. The present research has not yet quantified the influence of the particle size distribution of drill cuttings on the integrity of sampling. The influence mechanism of the shape of the drill bit on the migration of coal cuttings is still not precise. Furthermore, bit geometry effects on cutting migration behavior have received limited research attention. This study employs the CFD-DEM methodology to investigate the transport of cuttings following Rosin-Rammler particle size distributions during borehole sampling operations. Subsequent analysis quantifies airflow fields, particulate trajectories, and time-averaged transport velocities for discrete size fractions at axial/radial positions under variable rotational speeds.

## Materials and methods

### Mathematical equations

#### Gas governing equations.

In the CFD-DEM simulation process, the gas phase is a continuous phase that conforms to the following mass equation and momentum conservation equation [40]:


$$\frac{\partial }{\partial t}\left(\alpha_{f}\rho_{f}\right)+\nabla \cdot \left(\alpha_{f}\rho_{f}𝐮\right)=0$$ (1)

$$\frac{\partial }{\partial t}\left(\alpha_{f}\rho_{f}𝐮\right)+\nabla \cdot \left(\alpha_{f}\rho_{f}uu\right)=-\alpha_{f}\nabla P+\nabla \cdot \left(\alpha_{f}{𝕋}_{f}\right)+\alpha_{f}\rho_{f}𝐠+{𝐅}_{p\to f}$$ (2)

In these equations, t is time, $\alpha_{f}$ represents the volume fraction of the fluid, $\rho_{f}$ is the fluid density, 𝐮 is the velocity vector of the fluid phase, P represents pressure, 𝐠 is the gravitational acceleration vector, ${𝐅}_{p\to f}$ is the force exerted by the particles on the fluid, ${𝕋}_{f}$ is the stress tensor of the fluid phase, and the definitions are as follows:


$${𝕋}_{f}=\mu_{f}\left(\nabla 𝐮+\nabla {𝐮}^{T}\right)+\left(\lambda_{f}-\frac{\text{2}}{\text{3}}\mu_{f}\right)\nabla \cdot 𝐮𝕀$$ (3)

In the equation, 𝕀 is the unit tensor, using a realizable turbulence model to describe the gas flow inside the borehole. Due to the interaction between the random path and the fluctuating turbulent velocity field of the drilling cuttings generated at the bottom of the borehole, turbulence dissipation needs to be considered. The empirical formula for turbulent flow energy k is:


$$k=\frac{\text{3}}{\text{2}}I^{2}u_{i}^{2}$$ (4)

In the above formula, u is the average fluid velocity, m/s, and I is the turbulence intensity, defined as follows:


$$I=0.16(Re\mathrm{\backslash nolimits}{)}^{\frac{\text{1}}{\text{8}}}$$ (5)

In the above formula, Re\nolimits is the Reynolds number. Turbulent flow energy dissipation rate ε at the inlet is:


$$\varepsilon ={\sqrt[{4}]{C_{\mu }^{3}}}^{2}\frac{\sqrt{k^{3}}}{l}$$ (6)

In the above equation, $C_{\mu }^{3}$ is an empirical constant, l is the turbulence characteristic length, m, and the definition is as follows:


$$l=0.07d_{e}$$ (7)

In the above equation, $d_{e}$ is the characteristic length

#### Discrete phase governing equations.

The discrete phase also follows the conservation equation, and the momentum conservation equation for DEM particles with time t is [40]:


$$m_{p}\frac{d{𝐯}_{p}}{dt}=+{𝐅}_{c}+{𝐅}_{f\to p}+m_{p}𝐠$$ (8)

**Fig 2:**
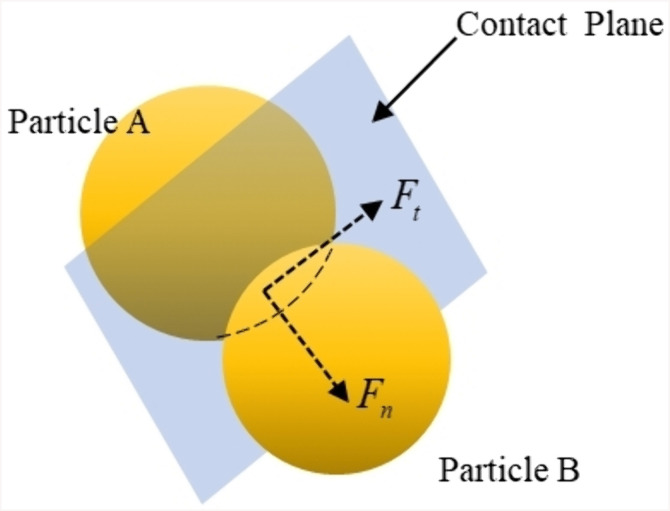
Particle to particle contact model.

Among them, ${𝐯}_{p}$ represents the instantaneous velocity, ${𝐅}_{c}$ is the contact force between particles or between particles and walls, and ${𝐅}_{f\to p}$ is the force exerted by the fluid on the particles. The contact force model uses the Hertz-Mindlin no-slip free model, and the inter-particle contact model is shown in Fig 2.

The contact force between particles or between particles and walls, ${𝐅}_{c}$ is defined as follows:


$${𝐅}_{c}=F_{n}𝐧+F_{t}𝐭$$ (9)

Where $F_{n}$, $F_{t}$ are the amplitudes of the normal and tangential components, respectively. In addition, the conservation equation of angular momentum for DEM particles is:


$$I_{p}\frac{d\omega_{p}}{dt}=M_{b}+M_{c}$$ (10)

$${𝕁}_{p}\frac{d\omega_{p}}{dt}={𝐌}_{c}+{𝐌}_{f\to p}$$ (11)

Where ${𝕁}_{p}$ is the particle’s moment of inertia, $\omega_{p}$ is the particle’s angular velocity, and ${𝐌}_{c}$ is the net torque generated by the tangential force that causes the particle to rotate.

**Fig 3:**
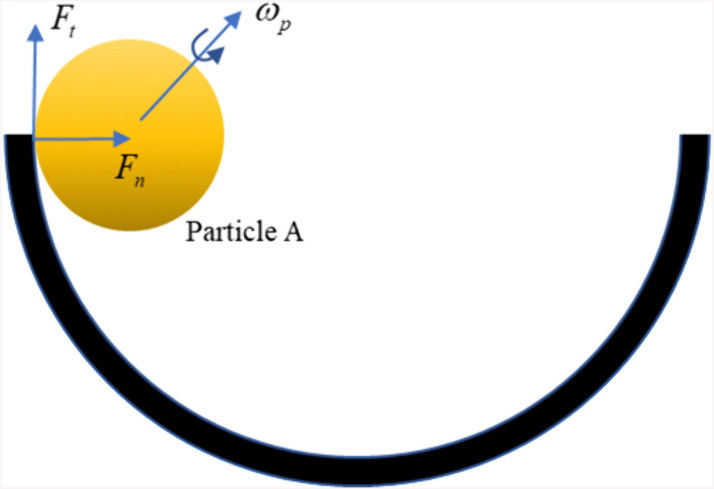
Friction model between the wall and particle A.

The frictional force of particles in the borehole is shown in Fig 3.

Equation for the rolling resistance torque acting on the particles:


$${𝐌}_{r}=-\mu_{r}|𝐫|F_{n}\frac{\omega }{\left|\omega \right|}$$ (12)

Where $\mu_{r}$ is the rolling resistance coefficient, ω is the particle angular velocity vector, and the direction of the rolling resistance vector will be the same as the direction of that angular velocity, |𝐫| is the particle rolling radius, 𝐫 is the vector connecting the particle center of mass to the contact point.

#### Inter-phase force equations.

The inter-phase force contains the force of the fluid on the particle phase and the force of the particles, relative to the fluid as the force generated by the fluid on the particles, defined as follows [40]:


$${𝐅}_{p\to f}=-\frac{\sum {\mathrm{\backslash nolimits}}_{p=1}^{N}{𝐅}_{f\to p}}{V_{c}}$$ (13)

Where, N is the number of particles in the grid, $V_{c}$ is the fluid grid volume, and ${𝐅}_{f\to p}$ is the force generated by the fluid on the particles, mainly including drag force, pressure gradient force, virtual mass force, and lifting force. The fluid force on the particles is defined as:


$${𝐅}_{f\to p}={𝐅}_{D}+{𝐅}_{\nabla p}+{𝐅}_{L}+{𝐅}_{VM}+{𝐅}_{others}$$ (14)

Among them, ${𝐅}_{D}$ is the drag force,${𝐅}_{\nabla p}$ is the pressure gradient force,${𝐅}_{L}$ is the lift force, ${𝐅}_{VM}$ is the virtual mass force, ${𝐅}_{others}$ is other acting forces on particles. Since particles are mainly affected by trailing and pressure gradient forces during transport, the ${𝐅}_{f\to p}$ can be simplified as:


$${𝐅}_{f\to p}={𝐅}_{D}+{𝐅}_{\nabla p}$$ (15)

Where the drag force on particles ${𝐅}_{D}$ is defined as:


$${𝐅}_{D}=\frac{\text{1}}{\text{2}}C_{d}\rho_{f}A_{p}\left|v_{s}\right|v_{s}$$ (16)

Where $C_{d}$ is the drag force coefficient, $\rho_{f}$ is the continuous phase density, $A_{p}$ is the projected area of the particles, $v_{s}$ is the particle slip velocity, which is defined as follows:


$$v_{s}=v-v_{p}$$ (17)

Where v denotes the instantaneous velocity of the continuous phase, the particle drag coefficient correlates intrinsically with flow regime characteristics. Given the dense-phase flow conditions governing particulate motion in the borehole, scholars have developed multiple drag coefficient models to quantify interphase momentum transfer. This investigation adopts the Gidaspow-Bezburuah-Ding drag model [41,42], validated for high-density particulate systems. The drag coefficient is mathematically defined as follows:


$$C_{d}=\{\begin{array}{cc} {}*20l\frac{24}{\alpha_{f}Re_{p}}[1+0.15{\left(\alpha_{f}Re_{p}\right)}^{0.687}]{\alpha_{f}}^{-1.65} & \alpha_{f}>0.8;\alpha_{f}Re_{p}<1000\\ 0.44{\alpha_{f}}^{-1.65} & \alpha_{f}>0.8;\alpha_{f}Re_{p}\geq 1000\\ 200\frac{\alpha_{f}}{\alpha_{f}\phi^{2}Re_{p}}+\frac{\text{7}}{3\phi } & \alpha_{f}\leq 0.8 \end{array}$$ (18)

Where, $\alpha_{f}$ is the fluid volume fraction, $Re_{p}$ is the particle Reynolds number, and ϕ is the particle sphericity.

### CFD-DEM coupling method

**Fig 4:**
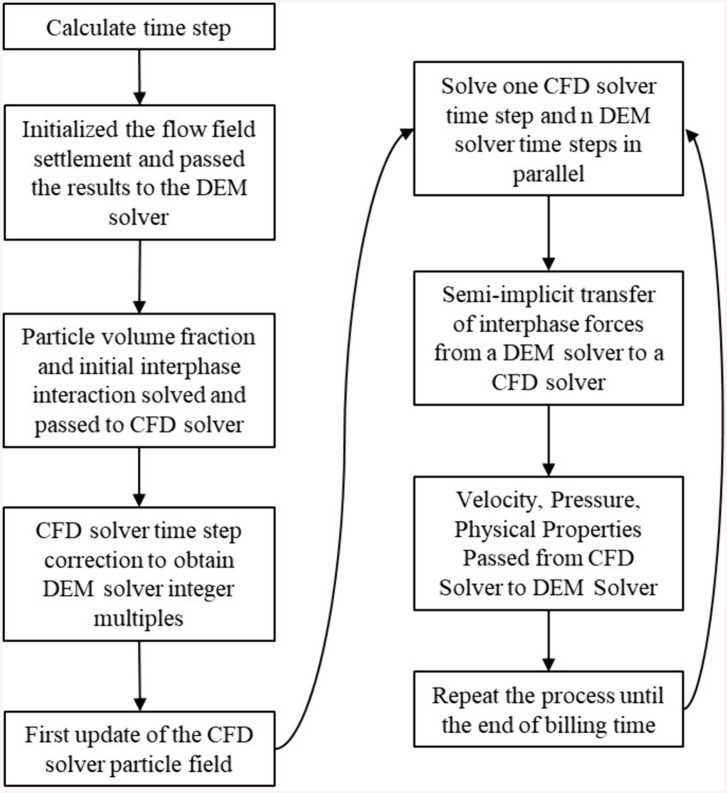
CFD-DEM coupled solution flowchart.

The CFD-DEM coupled methodology differs fundamentally from standalone CFD flow field computations or discrete particle field simulations. Bidirectional data exchange occurs between solvers: the CFD module transmits fluid phase velocity, pressure, and physical properties to the DEM module, while the DEM module transmits particulate volumetric concentration, position, and kinematic data to the CFD module. Fig 4 schematizes this solution framework workflow. [21,43].

Unlike alternative CFD-DEM coupling methodologies, the Fluent-Rocky DEM platform implements a heterogeneous computing architecture: the DEM solver executes on a GPU while the CFD solver operates on a CPU. This parallelized framework with solver synchronization significantly accelerates computational throughput in coupled simulations.

### Validation method

To quantitatively validate the accuracy of the fluid flow field simulation, a comparison based on the Reynolds number (Re\nolimits) and the Darcy friction factor (f) was performed. The hydraulic diameter (Dh) of the annulus and the average air velocity were used to calculate Re. The pressure drop (ΔP) along the borehole length L obtained from the simulation was used to calculate the simulated friction factor (f_sim) according to the following equation:


$$\Delta P=f\_sim\cdot (L/Dh)\cdot (\rho \cdot V^{\text{2}}/\text{2})$$ (19)

The calculated pairs of (Re\nolimits, f_sim) from various operational conditions were then compared against the well-established empirical correlation for turbulent flow in smooth pipes, the Blasius formula.

### Models

In this numerical investigation, the following assumptions were made [36,40]:

1) The coal seam gas is treated as an ideal gas.
2) The shapes of drill cuttings are treated as spherical, and their particle size distribution follows the Rosin-Rammler distribution function.
3) Mass transfer of drill cutting particles to the surface of the fluid is neglected.
4) The rotational axis of the drill rod is assumed to be centered within the drill rod’s axis, with no eccentric rotation.

#### Geometric model.

**Fig 5:**
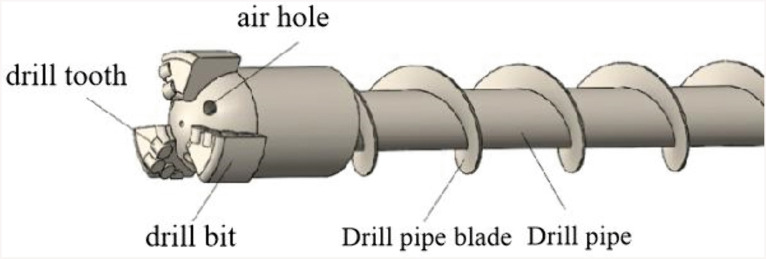
Geometrical modelling of drill rod and drill bits.

A SolidWorks-based geometric model of pneumatic sampling rods and bits was constructed per actual configurations in Fig 5. The three-wing PDC bit features internal helical blade channels. High-pressure gas propagates from the borehole orifice to the base via the rod’s central conduit, transporting bit-generated cuttings upward through the tool-wall annulus. A 5 mm basal clearance zone accommodates multiphase flow. Computational constraints necessitated simplification of bit-top cutters and auxiliary vent ports while retaining wing geometry fidelity. Critical dimensions include the following: bit length is 0.15 m, rod length is 1 m, and borehole length is 1.17 m.

Grid independence verification required pressure monitoring at four axial stations within the rod-wall annulus spanning the borehole orifice to base, supplemented by a transverse monitoring plane orthogonal to the rod axis.

The specific parameters of the drill rods and drill bits used in the simulation are presented in Table 1.

**Table 1:** Drill rod and drill bit parameters.

Parameter name	Value	Parameter name	Value
Outside diameter of drill rod	0.073m	Types of drills	3-bit PDC
Inner diameter of drill rod	0.04m	Drill outer diameter	0.094m
Spiral blade width	0.005m	Air pore angle	45°
Blade height	0.0105m	bit spacing angle	120°
Pitch of spiral	0.1m	Drill bit spacing angle	120°

**Fig 6:**
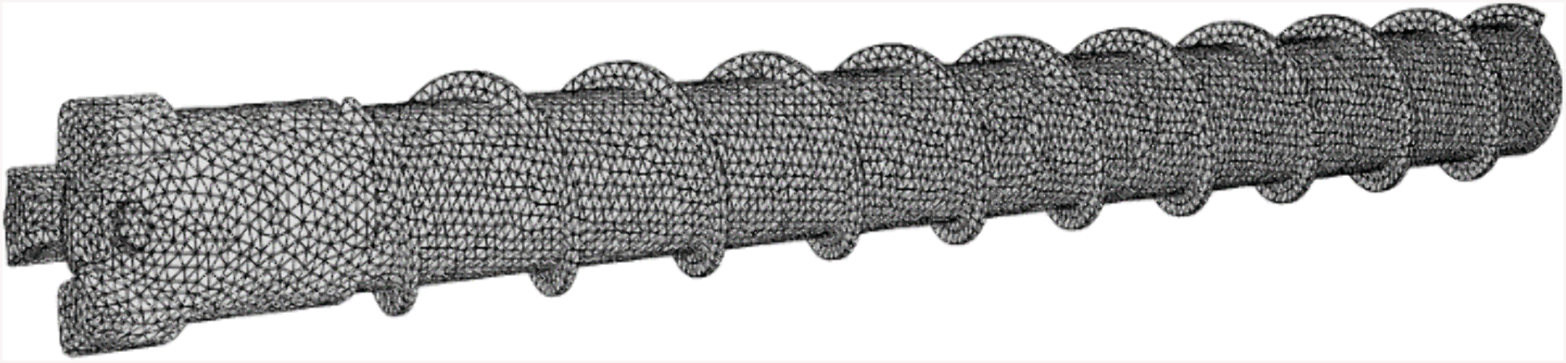
The geometry mesh effect of the drill rod and bit.

Mesh refinement enhances solution accuracy at the expense of computational efficiency. When particulate dimensions approach or exceed local cell size, solution fidelity degrades. Consequently, a minimal sufficient mesh density was implemented to preserve accuracy. Three discretization schemes were generated in ANSYS Fluent: fine (327,366 cells), medium (175,647 cells), and coarse (136,069 cells). Pressure gradient profiles along the borehole monitoring line P1-P2 across these schemes validated solution invariance. The medium-resolution topology is depicted in Fig 6.

Grid independence verification required four axial pressure monitoring lines spanning the borehole orifice to base within the rod-wall annulus, complemented by a transverse monitoring plane orthogonal to the rod axis.

#### Simulation parameters setup.

The air flow speed at the drill rod inlet was set as 20 m/s, with the gas treated to be ideal air, and the energy equation was not considered. The direction of gravity was defined as the negative direction of the z-axis. The pressure-velocity coupling was implemented using the phase-coupled SIMPLE algorithm with PRESTO!. Scheme for pressure interpolation. A first-order upwind scheme was employed for momentum and volume fraction equations, while second-order upwind discretisation was applied to the turbulence kinetic energy and dissipation rate transport equations. For the calculation of momentum and volume fractions, one section of the upwind format was selected, while turbulence kinetic energy and turbulence dissipation rate were treated in the second-order upwind format. In the coupled CFD-DEM approach, the continuous and discrete phases were solved in a staggered manner, requiring careful selection of time step sizes. The DEM time step was determined according to the Rayleigh wave propagation time criterion for particle contacts.

The boundary materials in the drilling simulation consist of drill cuttings, borehole wall (coal formation), and drill rod. The density, Poisson’s ratio, and shear modulus of the drill rod and coal are listed in Table 2.

**Table 2:** Material parameters.

Materials name (Unit)	Coal	Steel
Density (kg/m^3^)	1400	7800
Poisson’s ratio	0.3	0.3
Shear modulus (Pa)	1e + 8	1e + 10

The Hertz-Mindlin contact model was implemented to characterise particle-particle, particle-coal, and particle-drill rod interactions. The corresponding contact mechanical parameters are summarised in Table 3.

**Table 3:** Contact parameters.

Parameter name	Coal-Coal	Cuttings-steel	Cuttings-Coal
Coefficient of restitution	0.5	0.5	0.5
Coefficient of static friction	0.6	0.4	0.6
Coefficient of rolling friction	0.05	0.05	0.05

#### Drill cuttings production.

The particle size distribution of drill cuttings produced during the drilling process is described by the Rosin-Rammler distribution function [44,45]:


$$F(d)=1-\exp(-(\frac{d}{d_{ref}}{)}^{q})$$ (20)

**Fig 7:**
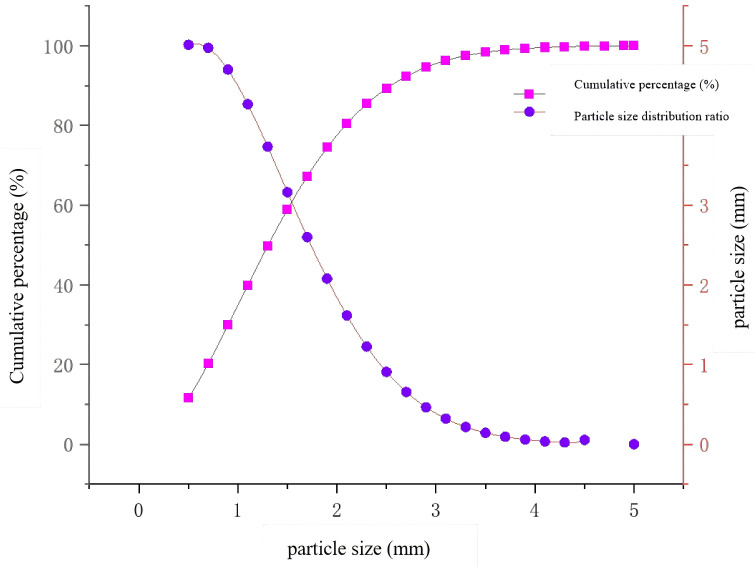
Drill cuttings particle size distribution.

Where q is the distribution index, $d_{ref}$ is the particle reference size, with minimum and maximum particle diameters set to 0.5 mm and 5 mm, the reference particle size was assigned 1.5 mm, the value of the distribution index q is 1.8. The generated particle size distribution of coal cuttings in the borehole is shown in Fig 7.

The mass production rate at the bottom of the borehole was set to 0.03 kg s^-1^ (30g/s). The feed speed of the drill bit v was maintained at 0.005m s^-1^(5 mm/s). The rotational speed of the drill rod was set to be 0, 50, 100, 150, and 200 rpm, respectively. For numerical stability, the Fluent solver utilized a fixed time step of 0.001 s, while the DEM time step in Rocky DEM was dynamically adjusted through its built-in particle contact resolution algorithm.

## Results

### Grid independence verification and validation

**Fig 8:**
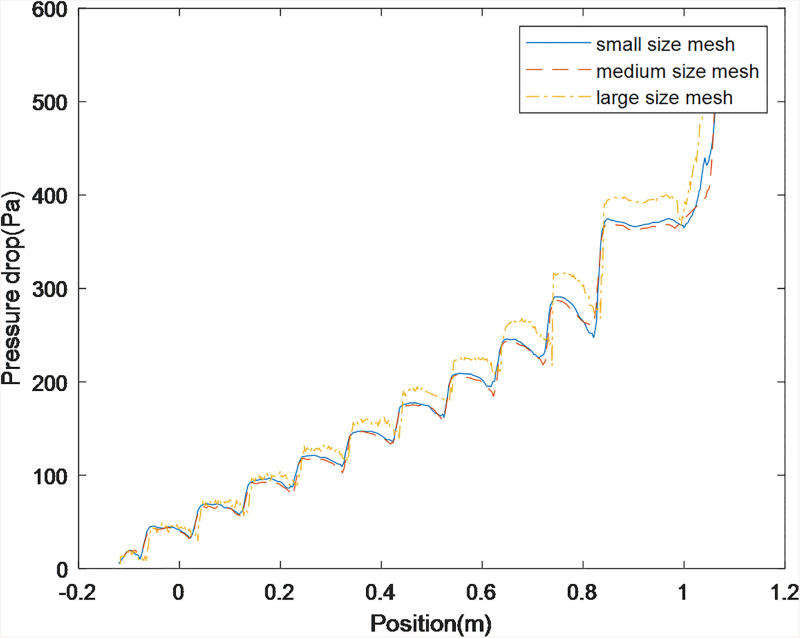
Grid independence test result.

Grid independence verification was conducted by monitoring pressure drop along a linear measurement path using progressively refined meshes. Fig 8 presents pressure differential data across discretization schemes. Results conclusively demonstrate grid convergence at medium resolution, therefore, this discretisation level was implemented in subsequent simulations.

Based on the calculation method in the validation method section and the parameters in the “Simulation parameters setup” section, the pressure drop in this article is calculated. During the calculation process, the values of the Validation parameters used are as shown in Table 4.

**Table 4:** Validation parameter value.

Name of parameter	Value	Name of parameter	value
Outside diameter of drill rod	0.073m (73 mm)	Drill outer diameter	0.094m(94 mm)
Inner diameter of drill rod	0.04 m (40 mm)	Hydraulic diameter	0.0021m
Spiral blade width	0.005m (5 mm)	Calculate the length of the drill hole	1m
Blade height	0.0105m(10.5 mm)	Inlet velocity	20m s^-1^

The Reynolds number Re\nolimits obtained based on the above parameters is about 28,583. This indicates that the k-e turbulence model selected in this paper is accurate. The theoretical pressure drop of smooth drilling holes, calculated using the Blasius friction factor formula, is approximately 282 Pa/m. The simulated pressure drop result is 370 Pa m^-1^. Due to the irregularity of the drill bit size and its being larger than the outer diameter of the drill pipe, compared with the drilling under smooth conditions, the pressure drop value will increase. The simulated pressure drop result is of the same order of magnitude as the calculated pressure drop, which is a reasonable value.

### Gas phase

**Fig 9:**
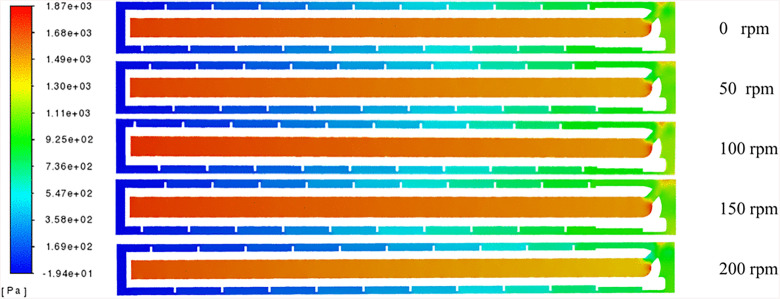
Static pressure distribution in the borehole at different rotational speeds.

Fig 9 presents the hydrodynamic pressure distribution within the borehole flow field under varying drill rod rotational speeds (0–200 rpm). The flow field can be divided into distinct pressure regions: a decreasing pressure zone extending from the inlet to the drill bit, low-pressure zones within the pneumatic channels of the drill bit, and high-pressure zones within the annular space of the borehole. In Addition, a progressive pressure decrease is observed along the annular space between the drill rod and the borehole wall. Variations in drill rod rotational speed induce fluctuations in the surrounding pressure zones. Furthermore, a pressure differential is detected between the drill rod wall and the rod body on either side of the blades, which is attributed to the inflow pattern created by the drill rod blades. This configuration channels the downhole pressure airflow and drill cuttings through the inter-blade pathways into the borehole.

**Fig 10:**
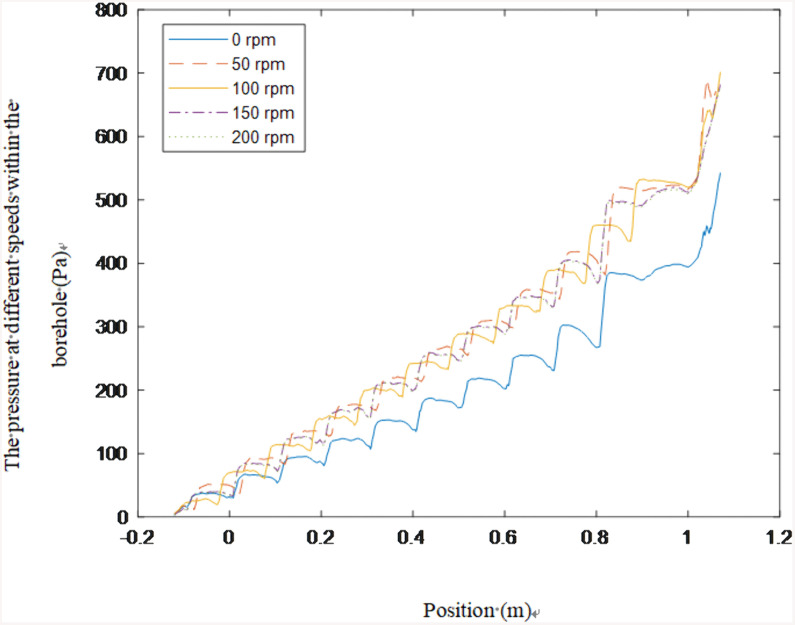
Pressure values for different positions in the borehole.

The design of the helical drill rod, combined with the drill bit geometry and blade configuration, induces differential flow fields within the borehole. As the rod rotates, both axial and radial velocities of borehole gas flow increase. Fig 10 illustrates pressure curves recorded during rotational speed testing. When stationary, the borehole exhibits slight pressure attenuation. During rotation initiation, pressure progressively decreases from the borehole bottom toward the orifice. Notably, each gas passage through the restricted clearance between rod blades and borehole wall induces significant pressure depression. Subsequently, as gas flows past this constriction, pressure recovers through flow area expansion, leading to increased flow velocity.

### Particle phase

**Fig 11:**
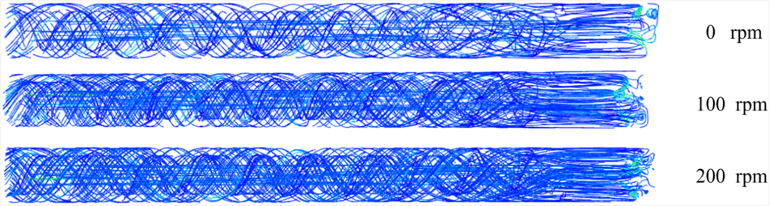
Drill cuttings transportation characteristics around the drill rod in the borehole.

Fig 11 Characterizes borehole cuttings transport dynamics under varying rotational speeds. The bladed rod system (combining helical blades and bit geometry) creates non-axisymmetric flow patterns distinct from non-bladed rod configurations. Blade-induced vorticity entrains particulate matter into helical trajectories. Additionally, subterranean-generated drill cuttings migrate upward through inter-blade clearance pathways, ultimately discharging at the borehole orifice.

The three-wing PDC bit configuration governs cuttings distribution patterns at the borehole base. Specifically, particulate ingress predominates through inter-wing clearances compared to on-wing accumulation. This phenomenon stems from bit-rod coupling dynamics and rotational periodicity of inter-wing gaps about the central axis, inducing cyclical particulate density oscillations within the borehole. Consequently, coarse drill cuttings experience enhanced blade-proximity impedance during borehole ingress attempts.

## Discussions

### Flow streamlines characteristics

**Fig 12:**
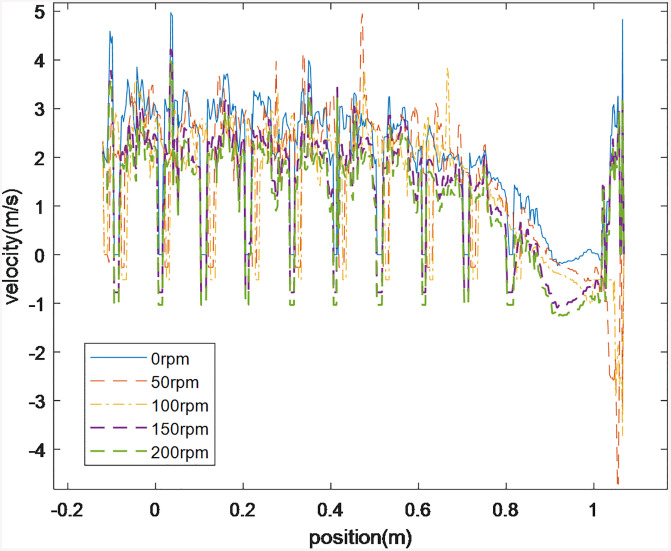
Gas phase flow lines in the borehole at different rotational speeds.

Fig 12 depicts flow streamlines within the borehole under varying operational speeds. The gas enters the bit face through the rod’s central channel, generating a localized pressure nucleus within its internal cavity. With increasing rod rotational speed, the peak pressure magnitude in this zone progressively amplifies, intensifying the vortical flow pattern in the borehole.

**Fig 13:**
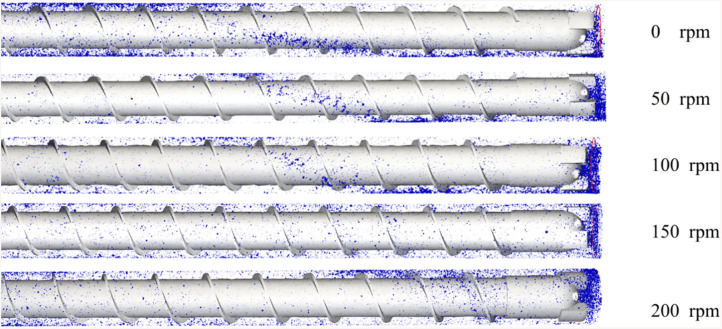
Axial velocity in the borehole at different rotational speeds.

Fig 13 depicts the cutting axial velocity profile at varying rod rotational speeds within the borehole, measured along a linear monitoring path. Axial velocity maintains a stable equilibrium in the overflow zone, exhibiting negligible velocity gradients. In contrast, pronounced velocity modulation occurs in high-flow-gradient regions. With increasing rotational speed, flow reversal emerges adjacent to the rod blades. Specifically, translational velocity vectors invert near the bit face, resulting from countercurrent airflow penetration through bit ports into the borehole base.

**Fig 14:**
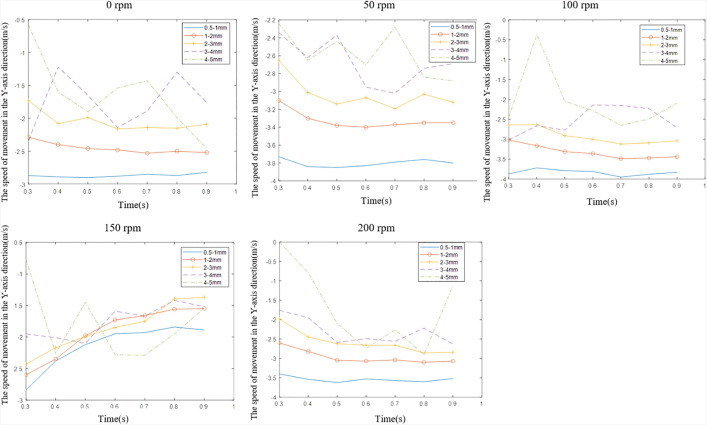
Drill cuttings transport characteristics around the drill rod in the borehole.

Rotational speed and particle size distribution significantly influence the cuttings’ axial migration velocity. Fig 14 depicts the temporal evolution of migration velocities for varying particle size ranges (0.5 mm-3 mm) during 0.3 s–1 s intervals and differential rotational speeds. Negative values represent the axial velocity of coal cuttings migration towards the drill hole. Experimental data demonstrate positive velocity scaling with rotational speed across all size fractions. For instance, 0.5 mm-1 mm drill cuttings exhibit velocity escalation from 2.5 m s^-1^ to 2.8 m s^-1^. In coal seam gas content determination via borehole sampling, bottom-generated cuttings migration velocity variability must be incorporated into lost gas content estimations.

### Effect of drill rod rotation speed on mass flow rate

**Fig 15:**
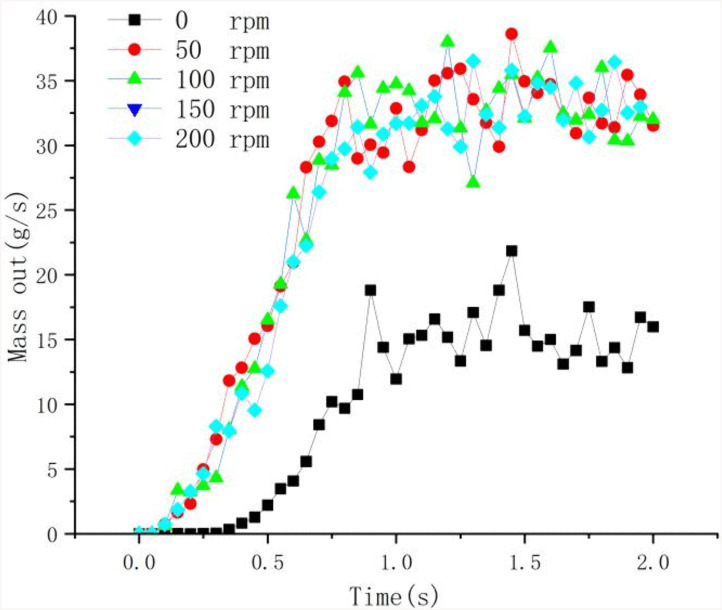
Mass outflow rate at the opening end of the borehole (g/s).

Fig 15 quantifies the cuttings mass out through the borehole orifice under varying rod rotational speeds. Experimental data reveal that post-0.8s temporal domain achieves quasi-steady cuttings discharge with superimposed stochastic fluctuations. Critical analysis shows that stationary rod conditions yield 50% mass flux reduction compared to rotational operation modes, demonstrating insufficient cuttings evacuation capacity of solo compressed air injection. The rotational mechanics prove essential for effective cuttings expulsion, confirming rotational enhancement in the efficiency of borehole cleaning.

### Influence on gas content measurement

As mentioned above, in the process of taking coal samples using air pressure to measure the gas content of the coal seam, the transport speed of coal cuttings inside the borehole varies. This results in coal samples obtained at the borehole mouth being collected at different times. Due to the difference in exposure time, this variation is not conducive to the accuracy of gas loss determination in the direct method for measuring coal seam gas content. Therefore, when selecting coal samples, adjacent mesh sizes of sieves should be used to obtain coal samples with similar exposure times, such as 3 mm and 4 mm sieves.

In this paper, the CFD-DEM method was used to conduct numerical simulations of the transport characteristics of coal cuttings during air pressure drilling coal sampling. The experimental results show that variations in the transport speed of different-sized coal cuttings particles inside the borehole compromise the accuracy of gas loss determination in the direct method for coal seam gas content measurement. Note that this paper only considers air pressure drilling scenarios. For longer boreholes, drilling employs the mixed slag discharge method. Therefore, further research is needed on mixed slag discharge conditions.

## Conclusions

This study employs CFD-DEM coupled methodology to investigate gas-particulate flow dynamics during pneumatic sampling operations, elucidating the mechanisms by which drill rod rotation speed, particle size distribution, and bit geometry influence gas sampling integrity. Key conclusions are as follows:

1) Fine drill cuttings (0.5 mm–1 mm) exhibit 100% higher axial migration velocity toward the borehole orifice compared to coarse cuttings (1 mm–2 mm) under 50 rpm operational conditions. Rotational speed intensification enhances cuttings axial transport, demonstrating velocity scaling from 2.8 m s^-1^ to 3.9 m s^-1^ for 0.5 mm–1 mm drill cuttings as speed elevates from static to 100 rpm. Optimized rotational thresholds exist where velocity maximization occurs. Particle size-dependent velocity differentials induce particle segregation during borehole transit, necessitating velocity stratification analysis in lost gas content estimations. Therefore, when selecting coal samples, adjacent mesh sizes of sieves should be used to obtain coal samples with similar exposure times, such as 3 mm and 4 mm sieves.
2) Bit geometry and rod configuration critically govern cuttings transport mechanisms. Bottom-generated drill cuttings traversing the bit structure become subject to wing-induced flow deflection, necessitating passage through inter-wing clearances to access the bit rear chamber. Concurrently, radial blade height imposes geometric constraints, preventing direct transit of coarse cuttings through blade-annulus interfaces. When sampling, one should select the drill pipe with a larger space between the various wings of the drill bit to increase the probability of large coal particles reaching the hole opening.
3) This article investigated only the gas-solid two-phase flow migration characteristics of drill cuttings during the dry rotary drilling process. In the future, research should be extended to the gas-solid two-phase flow migration characteristics under air-water mixture slag discharge conditions and for non-spherical coal particles.
4) It should be noted that the factors such as truncation error, round-off error, and model error still exist between numerical simulation and direct experimental research, which may affect the experimental results. In future research, we will continue to carry out experimental studies to avoid the limitations of using only numerical simulation methods.
